# Sellar spine: A rare Bony variant of the Sella Turcica

**DOI:** 10.4102/sajr.v26i1.2371

**Published:** 2022-04-28

**Authors:** Luke D. Metelo-Liquito, Thandi E. Buthelezi

**Affiliations:** 1 Department of Diagnostic Radiology, Faculty of Health Sciences, University of the Witwatersrand, Johannesburg, South Africa

**Keywords:** sellar spine, pituitary fossa, sella turcica, bony variant, hypoprolactinaemia

## Abstract

A sellar spine is a rare osseous projection from the dorsum sellae, resulting in variable compression of sellar and suprasellar structures and varied clinical presentations. CT is the diagnostic modality of choice, while variable signal intensity on MRI may mimic a pituitary microadenoma. A patient presented with hypoprolactinaemia and puerperal alactogenesis due to a sellar spine diagnosed on CT Brain. Neurosurgical and endocrine review and pituitary MRI were recommended with subsequent loss to follow-up.

## Introduction

A sellar spine is an osseous spur projecting from the dorsum sellae and is a rare variant of the pituitary fossa. This reported case of a sellar spine is unique for two reasons. To our knowledge, this is the first reported sellar spine that arises from the mid-ventral aspect of the dorsum sellae, with most of the previously described cases in the literature arising from the inferior ventral aspect of the dorsum sellae and two arising from the superior aspect of the dorsum sellae.^1,2,3,4,5,6,7,8,9,10^ Secondly, the history of puerpural alactogenesis and mild hypoprolactinaemia has not yet been described in any of the known reported cases in the literature.

## Patient presentation

A 37-year-old woman with no known co-morbidities presented with a single episode of menorrhagia (1-month history of menstrual bleeding), severe constant headaches for one year, and a history of puerperal alactogenesis in all four of her previous pregnancies. Physical examination was unremarkable. Hormone levels: Thyroid-stimulating hormone (TSH) and prolactin were mildly decreased. Follicle-stimulating hormone (FSH) and Luteinizing hormone (LH) were elevated (TSH: 0.33 mIU/L (normal range 0.35–5.50 mIU/L), thyroxine (free T4): 17.5 pmol/L (normal reference range 11.5–22.7 pmol/L), prolactin: 4.7 µg/L (normal range 4.8–23.3 µg/L), FSH: 31 IU/L, LH: 7.7 IU/L, oestradiol: < 19 pmol/L, progesterone: < 0.2 nmol/L). Pelvic ultrasound was unremarkable. She was referred to the radiology department for CT Brain to exclude any pituitary pathology.

**FIGURE 1:**
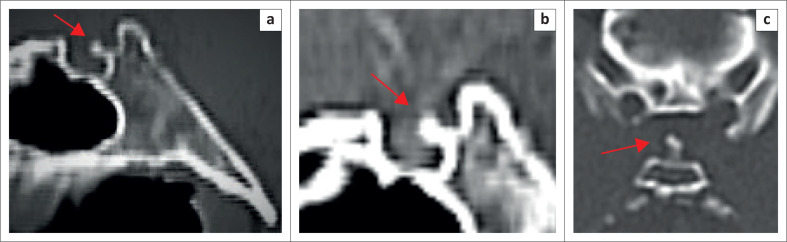
CT of the pituitary fossa. (a) Bone window in the sagittal plane in the midline demonstrating the sellar spine (arrow) protruding into the pituitary fossa producing a ‘figure of 3’ sign of the dorsum sellae. (b) Soft tissue window in the sagittal plane in the midline demonstrating compression of the pituitary and distal pituitary stalk by the sellar spine (arrow). (c) Bone window in the axial plane demonstrating the midline position of the sellar spine (arrow).

**FIGURE 2:**
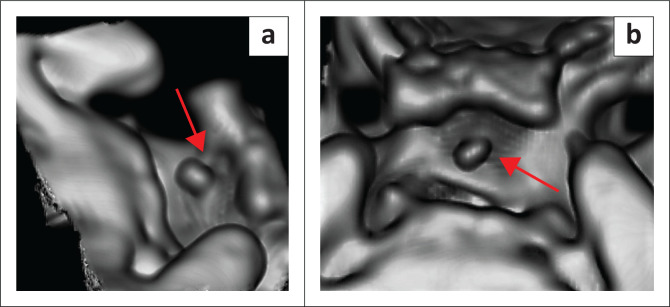
Volume rendered CT of the sella turcica, (a) oblique view and (b) superior view, demonstrating the sellar spine (arrow) protruding into the pituitary fossa.

CT Brain demonstrated a bony spur, consisting of a narrow stalk with a mildly distended tip, arising from the mid-anterior aspect of the dorsum sellae in the midline, projecting anterosuperiorly into the pituitary fossa. The spur measured 5.5 mm in length and compressed the posterior pituitary and the distal posterior aspect of the pituitary stalk (Figures 1 and 2). No other intracranial pathology was noted.

Non-urgent neurosurgical review, repeat endocrine profile and MRI of the pituitary gland with dynamic contrast enhancement were recommended; however, the patient was lost to follow-up.

## Discussion

A sellar spine is a rare midline osseous spur, which arises from the dorsum sellae and projects into the pituitary fossa.^1^ In 1977, Lang et al. described the first case in an article describing atypical ossifications of the sella turcica.^11^ It is a rare entity with an estimated incidence of 1 in 5000–8000.^1,12^

The leading hypothesis for its development is that it is due to the failure of regression of the most cephalic aspect of the notochord through the clivus into the vertebral column, resulting in a remnant of a notochordal rest in the fetal sella; this remnant then ossifies.^1^ Other proposed hypotheses by Dietemann et al. include ossification of a dural fold and an ossified vascular channel.^1^

In all the described cases, the sellar spine was located in the midline in keeping with the foremost theory that it is an ossified remnant of the notochord.^1,2,3,4,5,6,7,8,9,10,11,12,13,14,15,16^ The most consistent imaging morphology of the bony spur among the literature was that of a narrow cylindrical bony stalk with a mildly distended tip and a flattened triangular base in continuity with the dorsum sellae.^1,2,3,9,11,12,13^ In most cases, the tip had a smooth contour; however, in one of the cases, it was irregular.^1,2,3,7,11,12^ The maximum described length was 9.0 mm.^2^ For the remainder of the reported cases, the length of the sellar spine ranged between 3.8 mm and 5.0 mm, making this case the second longest reported case at 5.5 mm.^3,4.5,11,12,14^ In most of the cases, the sellar spine arose from the inferior aspect of the anterior dorsum sellae with only two cases reported to be arising from the superior aspect.^1,2,3,4,5,6,7,8,9,10^ To our knowledge, this is the first case, reported, where the sellar spine arises from the mid-anterior aspect giving a ‘figure of 3’ sign of the dorsum sellae in the sagittal plane (Figure 1). The spine most commonly projects in an anterosuperior orientation, with one case observed to be in a more horizontal orientation and one case in an anteroinferior orientation.^2^

The most commonly compressed structure is the posterior pituitary, in keeping with the anteroinferior origin from the dorsum sellae.^8^ In the two cases where there was an anterosuperior origin from the dorsum sellae, compression of the pituitary stalk as well as the optic chiasm was observed.^5,6^ Our case compresses the junction of the stalk and posterior pituitary due to its mid-anterior origin.

The age at presentation is varied and ranges from 8 to 53 years, with most of the cases reported between the ages of 13 and 29 years.^1,2,3,4,5,6,7,8,9,10,11,12,13,14,15,16^ A possible explanation for this distribution is the slow progressive growth of the sellar spine over time, as well as normal progressive enlargement of the pituitary gland. This combination increases the probability of compression of the pituitary gland and thus the development of symptoms. This theory is supported by the findings of Hosokawa et al. who demonstrated interval growth of the sellar spine in a patient for whom serial images were obtained over time.^14^ On the contrary, Chivukula et al. demonstrated no significant growth on serial images obtained from their patient.^8^ Another possible explanation for the development of symptoms in our case is the normal growth of the pituitary gland in pregnancy, as postulated by Chivukula et al.^8^

The spectrum of described clinical presentations is varied, with most cases being asymptomatic.^7^ The most common presenting complaint among the reviewed literature was headache.^4,6,7,8,13,15^ The other common neurological manifestation was bitemporal hemianopia due to optic chiasm involvement.^8,9,15^

Of the endocrine manifestations, there were varying degrees of hypopituitarism, with diabetes insipidus being the most explicable of these findings.^2,9,15^ Few of the cases demonstrated pituitary hyperfunction, including hyperprolactinaemia and Cushing’s syndrome; the pathophysiological mechanism for this is not understood.^3,8,12^ Our case describes the first presentation with puerperal alactogenesis and mild hypoprolactinaemia. Other endocrine disturbances in our case included elevated FSH and LH levels and decreased TSH levels; this combination of findings has not been previously described and may be attributed to the combination of pituitary and pituitary stalk compression or laboratory error. A repeat hormonal profile would have been of great benefit had the patient not been lost to follow-up.

CT is the gold standard imaging modality to assess for a sellar spine as it appears as a well-ossified structure in continuity with the dorsum sellae.^7^ On MRI, sellar spines have variable signal intensity depending on the marrow content.^3,4,5,6,7,8,10,13,14,15,16^ In the absence of marrow, they are hypointense on T1- and T2-weighted images due to densely packed cortical bone and hyperintense on T1 and T2-weighted images if bone marrow is present; in addition, they are nonenhancing.^3,4,5,6,7,8,10,13,14,15,16^ A sellar spine is most commonly seen as a T1 hypointensity in the posterior pituitary and can thus be easily misdiagnosed as a pituitary microadenoma.^7^ The pituitary bright spot may be lost, and there may be deformation of the pituitary gland as adjunctive findings.^15^

Chivukula et al. described transsphenoidal resection of the sellar spine in their patient with panhypopituitarism.^8^ There was a reported complete resolution of symptoms and hormonal imbalance following the surgical resection of the sellar spine in their case.^8^

An important differential for an intrasellar bony projection is a post-traumatic base of skull fracture with a projection of a bony fragment into the pituitary fossa such as in the case described by Parizel et al.^17^

## Conclusion

Although a sellar spine is a rare entity, it should be considered in patients being referred for diagnostic imaging for potential pituitary pathology as they can present with variable symptoms and endocrine disturbances. CT is the imaging modality of choice because of its accurate visualisation of osseous pathology, while, caution should be practised when using MRI, as sellar spines can have variable signal intensity depending on the marrow content and can therefore mimic pituitary microadenomas.
